# Advancing Endocrine Disruptors via In Vitro Evaluation: Recognizing the Significance of the Organization for Economic Co-Operation and Development and United States Environmental Protection Agency Guidelines, Embracing New Assessment Methods, and the Urgent Need for a Comprehensive Battery of Tests

**DOI:** 10.3390/toxics12030183

**Published:** 2024-02-28

**Authors:** Sophie Fouyet, Marie-Caroline Ferger, Pascale Leproux, Patrice Rat, Mélody Dutot

**Affiliations:** 1 YSLAB, Recherche & Développement, 29000 Quimper, France; sophie.fouyet@yslab.fr; 2 CNRS CiTCoM, Université de Paris, 75006 Paris, Francepascale.leproux@u-paris.fr (P.L.); patrice.rat@u-paris.fr (P.R.)

**Keywords:** endocrine disruptor, alternative method, in vitro, validation, weight of evidence

## Abstract

Efforts are being made globally to improve the evaluation and understanding of endocrine-disrupting chemicals. Recognition of their impact on human health and the environment has stimulated attention and research in this field. Various stakeholders, including scientists, regulatory agencies, policymakers, and industry representatives, are collaborating to develop robust methodologies and guidelines for assessing these disruptors. A key aspect of these efforts is the development of standardized testing protocols and guidelines that aim to provide consistent and reliable methods for identifying and characterizing endocrine disruptors. When evaluating the potential endocrine-disrupting activity of chemicals, no single test is capable of detecting all relevant endocrine-disrupting agents. The test battery approach is designed to reduce the risk of false negative results for compounds with toxic potential. A weight-of-evidence approach is therefore necessary for endocrine disruptor evaluation. This approach considers various types of data from multiple sources, assessing the overall strength, consistency, and reliability of the evidence. OECD guidelines are highly regarded for their scientific rigor, transparency, and consensus-based development process. It is crucial to explore and develop new methodologies that can effectively evaluate the risks associated with potential endocrine disruptors. Integrating these methods into a comprehensive weight-of-evidence framework will enhance risk assessments and facilitate informed decisions regarding the regulation and management of these substances, ensuring the protection of human health and the environment from their adverse effects.

## 1. Introduction

An endocrine disruptor was defined by the World Health Organization (WHO) in 2002 as an exogenous substance or mixture that alters the function(s) of the endocrine system and consequently causes adverse health effects in an intact organism or its progeny or (sub)populations [1]. Today, there is a worldwide consensus on this definition. These effects can range from developmental abnormalities and reproductive disorders to neurobehavioral changes and cancer [2,3,4,5,6,7].

On 19 December 2022, the European Commission adopted the Delegated Act to introduce the new hazard classes under Regulation (EC) No 1272/2008 on hazard classification, labelling, and packaging of chemicals (CLP) [8]. The new hazard classes include endocrine disruptors for human health and the environment. The related Commission Delegated Regulation (EU) 2023/707 amending Regulation (EC) No 1272/2008 was published on 31 March 2023 [9]. Two categories of endocrine disruptors have been created: known or presumed endocrine disruptors (category 1) and suspected endocrine disruptors (category 2), both for human health and for the environment.

The classification in Category 1 is largely based on evidence from at least one of the following: human data, animal data, or non-animal data providing an equivalent predictive capacity as human or animal data. Such data should provide evidence that the substance meets all the following criteria: endocrine activity, an adverse effect in an intact organism or its offspring or future generations, and a biologically plausible link between the endocrine activity and the adverse effect. However, where there is information that raises serious doubt about the relevance of the adverse effects to humans, the classification in Category 2 may be more appropriate. A substance will be classified in Category 2 where all the following criteria are fulfilled: evidence of endocrine activity, an adverse effect in an intact organism or its offspring or future generations, and evidence of a biologically plausible link between endocrine activity and the adverse effect.

There are several tests used for the assessment of endocrine disruptors, which can be broadly categorized as follows:

- In vitro assays: These are tests performed in the laboratory using isolated cells or tissues to measure the effects of a substance on the endocrine system. In vitro assays cannot fully reflect a compound’s characteristics the way in vivo methods can, but they focus on specific mechanistic endpoints [10].
- In vivo assays: These tests involve exposing whole organisms to a substance to assess its effects on the endocrine system. Those tests are divided into mammalian tests, mostly in rodents, and non-mammalian tests (in fishes, amphibians, and birds) [10].
- Epidemiological studies can also be used when they investigate the relationship between exposure to a substance and changes in endocrine function in human populations [11].

Developing an in vitro assay to assess endocrine disruption can be challenging due to the complex nature of the endocrine system and the diverse mechanisms by which endocrine disruptors can cause adverse effects.

One of the main difficulties in developing an in vitro assay for endocrine disruption is identifying the relevant endpoints to measure. The endocrine system is involved in a wide range of physiological processes, and there are many different hormones and receptors that can be targeted by endocrine disruptors [12,13,14,15,16]. As a result, it can be difficult to determine which endpoints to measure in an in vitro assay to effectively capture the full range of endocrine disruption effects.

Another challenge is selecting appropriate cell lines and experimental models. The endocrine system is highly complex and involves multiple cell types and signaling pathways [17]. Therefore, choosing a relevant cell line or experimental model is critical to ensure that the in vitro assay accurately reflects the in vivo effects of endocrine disruptors. Additionally, the choice of cell culture conditions, such as the culture medium and growth factors, can also influence the results of the assay [18,19].

Assay sensitivity is another important consideration when developing an in vitro assay for endocrine disruption. Endocrine disruptors can have subtle effects on the endocrine system, and many standard cell-based assays may not be sensitive enough to detect these effects [20,21,22]. Therefore, it is important to optimize the assay conditions and endpoints to increase sensitivity and ensure that the assay can accurately detect even low levels of endocrine disruption.

Finally, the issue of assay validation is also critical. In vitro assays for endocrine disruption must be validated to ensure that they accurately predict the effects of endocrine disruptors in target species. Inter-laboratory validation studies are also important to ensure that the assay can be reliably replicated in different laboratories [23,24].

It is important to note that the specific tests used for the assessment of endocrine disruptors can vary depending on the regulatory agency.

The objective of this article is to review the in vitro methods to assess endocrine disruption, validated or under development, with respect to the new European regulation.

## 2. International Guidelines

The OECD (Organization for Economic Co-operation and Development) is an intergovernmental organization in which representatives of 38 industrialized countries in North and South America, Europe, and the Asia and Pacific region, as well as the European Commission, meet to coordinate and harmonize policies, discuss issues of mutual concern, and work together to respond to international problems. The OECD has developed a number of test guidelines and methods for the testing and assessment of chemical substances. The OECD guidance document 150 on standardized test guidelines for evaluating chemicals for endocrine disruption is intended to provide guidance for interpreting the outcome of individual tests and compiling evidence on whether or not a substance may be an endocrine disruptor [25]. The guidance document also provides a general description of each standardized test guideline and tabular presentations of the endpoints measured in each test and the endocrine pathway affected. The document also describes a Conceptual Framework for Testing and Assessment of Endocrine Disruptors that helps organize available test methods at different levels of biological organization to determine additional testing needs or conclude about the potential endocrine disruptor action of a chemical. The OECD guidelines and methods serve as a basis for mutual acceptance of data so that they are widely used by regulatory authorities and industry to assess the safety of chemicals. For example, the guidance for the identification of endocrine disruptors in the context of Regulations (EU) No 528/2012 concerning the making available on the market and use of biocidal products [26] and (EC) No 1107/2009 concerning the placing of plant protection products on the market [27] refers to the OECD guidelines (European Chemical Agency (ECHA) and European Food Safety Authority (EFSA) with the technical support of the Joint Research Centre [28].

The OPPTS (Office of Pollution Prevention and Toxics) is a division within the United States Environmental Protection Agency (EPA) that is responsible for developing and implementing policies, programs, and regulations related to pollution prevention and chemical management. The OPPTS has developed a number of guidelines that provide recommendations and guidance for conducting various types of environmental testing and risk assessment. The EPA OPPTS guidelines are developed specifically for use in the United States, and they are primarily focused on regulatory compliance under US law. The OPPTS guidelines are nevertheless widely used by regulatory agencies, industry, and researchers in the United States and around the world.

Overall, both the OECD and EPA OPPTS guidelines are important tools for ensuring that chemicals are evaluated in a consistent and scientifically rigorous manner and that appropriate risk management strategies are developed to protect human health and the environment. The in vitro OECD and OPPTS guidelines for the assessment of endocrine disruption can be classified according to the target mechanism.

## 3. Binding of a Substance to a Hormone Receptor

The ability of an endocrine disruptor to bind to a receptor depends on the chemical properties of the substance and the characteristics of the receptor, such as its shape, charge, and binding site [29]. Some endocrine disruptors have a chemical structure that resembles the natural hormone, allowing them to bind to the receptor in a similar way [30,31,32]. Diethylstilbestrol is a synthetic nonsteroidal estrogen, and it is probably the most famous endocrine disruptor [33,34,35]. Endocrine disruptors can also bind to the receptor and block the natural hormone from binding, resulting in a decrease in hormone activity [30,31,32]. These substances are known as antagonists. Last but not least, endocrine disruptors can also bind to the receptor and alter the way it interacts with other proteins or DNA, resulting in changes in gene expression or hormone signaling pathways. These substances are known as modulators [31,32,36].

The binding test that has been validated by the OECD is a biochemical in chemico test, which does not require the use of cells: Test No. 493: Performance-Based Test Guideline for Human Recombinant Estrogen Receptor (hrER) In vitro Assays to Detect Chemicals with Estrogen Receptor (ER) Binding Affinity (see Section 6).

Test No. 493 allows for the measurement of the ability of a radiolabeled ligand, [3H]-17β-estradiol (^3^H-E2), to bind with estrogen receptor alpha (ERα) in the presence of increasing concentrations of chemical tested (called a “competitor”) [37]. Chemical products tested with high ER binding affinity compete with the radiolabeled ligand at a lower concentration than compounds with lower affinity to the receptor. The assay includes a saturation binding experiment to establish the parameters of the receptor–ligand interaction, which is the first step in a series of molecular events that activate the transcription of target genes, resulting in a physiological change. This experiment allows us to characterize the number and binding affinity of receptors in a given batch in view of a second experiment, called the competitive binding experiment. This last one aims to determine the extent to which a tested chemical product competes with a radiolabeled ligand for binding to ERs. Specifically, it measures the binding to ER of ^3^H-E2 in fixed concentration in the presence of multiple concentrations of a tested chemical product. Quantitatively, it is possible to obtain the concentration of chemical product inducing half of the maximum inhibition of specific ^3^H-E2 binding, called IC50, as well as the relative binding affinities toward human recombinant ERα (hrERα) with the chemical’s product tested compared with E2. For this test, two methods are used whose principles are similar. The first is the Freyberger–Wilson (FW) assay, an estrogen receptor (ER) binding in vitro assay using an integral recombinant hERα. The second is the Chemical Evaluation and Research Institute (CERI, Tokyo, Japan) assay, an ER binding in vitro assay using the ligand binding domain of recombinant human ERα.

The OCSPP assay 890.1150 Androgen receptor binding assay identifies chemical substances that have the potential to interact with the androgen receptor (AR) in vitro (see Section 6) [38]. The Androgen Receptor Binding Assay identifies chemical substances that have the potential to interact with the AR in vitro. Androgens are sex hormones that play critical roles in male sexual differentiation, development, and maturation and also have some role in female development and physiology. Similar to the principle of the 493 test, it also consists of two sets of experiments: a saturation binding experiment and a competitive binding experiment. This assay measures the binding affinity of a substance to ARs by assessing its ability to displace the binding to AR of a radiolabeled reference substance, usually 5α-dihydrotestosterone (DHT) or a synthetic androgen R1881.

## 4. Transcriptional Activation/Inhibition Assays

**Figure 1:**
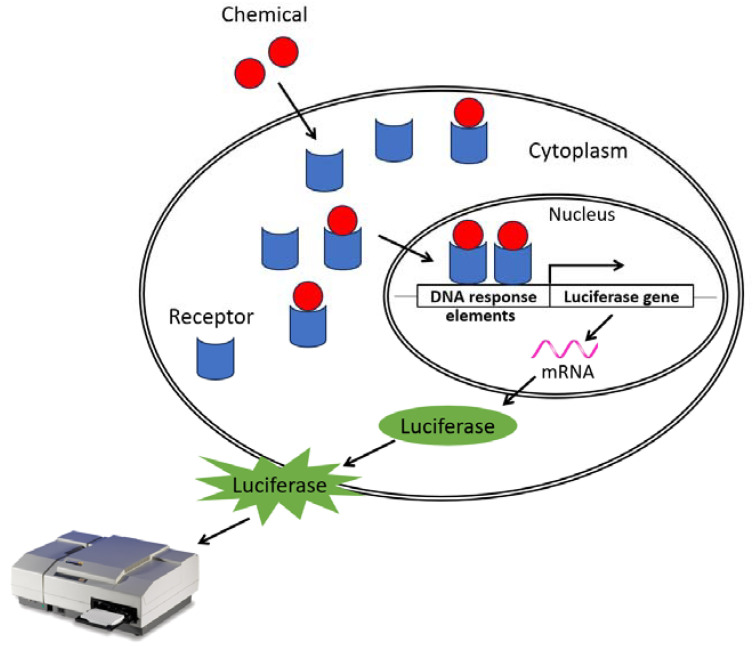
Formation of the receptor–ligand complex leading to DNA binding and, subsequently, light emission. In vitro tests are conducted using genetically modified cell lines expressing the luciferase gene to detect the transcriptional activation or inhibition activity on hormone receptors induced by a chemical.

The binding of a substance to a hormone receptor can also affect the transcriptional activity of the receptor. In order to evaluate the impact that these substances can have, in vitro tests based on the use of genetically modified cell lines expressing a reporter gene, firefly luciferase, have been developed and implemented. It works as follows: when a chemical binds to a receptor, the receptor–ligand complex undergoes translocation to the nucleus, where it binds to DNA response elements. This transactivates and induces the expression of luciferase that will transform luciferin (=substrate) into a bioluminescent product that can be measured quantitatively using a luminometer (see Figure 1). The molecules with an agonist effect increase the detected signal while the antagonist molecules decrease it. Thus, luciferase activity can be assessed quickly and inexpensively with the aid of many commercially available test kits.

These screening assays are therefore set up to evaluate the expression of genes regulated by nuclear receptors. Two families of assays have been validated by the OECD: Test Guideline No. 455: Performance-Based Test Guideline for Stably Transfected Transactivation In vitro Assays to Detect Estrogen Receptor Agonists and Antagonists [39] and Test Guideline No. 458: Stably Transfected Human Androgen Receptor Transcriptional Activation Assay for Detection of Androgenic Agonist and Antagonist Activity of Chemicals [40].

Test No. 455 detects ER receptor-mediated transactivation with chemiluminescence as a measurement parameter [39], and Test No. 458 detects androgenic receptor-mediated transactivation [40].

Test No. 455 uses three structurally and functionally similar in vitro assay methods to detect estrogen receptor (ERα and/or ERβ) agonist and antagonist substances:

- Stable transfection TA assay (STTA assay) using the hERα-HeLa-9903 cell line derived from immortalized human cervical cells and transfected with a hERα and a luciferase reporter gene to identify substances with estrogen agonist activity.
- VM7Luc ER TA assay using the VM7Luc-4E2 cell line derived from immortalized human adenocarcinoma (VM7) cells capable of expressing both types of estrogen receptors (primarily hERα and partially hErβ) endogenously. Then, they were stably transfected with the pGudLuc7.ERE plasmid to identify substances with estrogen agonist and antagonist activity.
- ERα CALUX using the ERα CALUX cell line derived from human osteosarcoma that expresses stably transfected human ERα. The assay is specifically designed to detect hERα-mediated transactivation.

Test No. 458 allows for the determination of AR agonist or antagonist substances with chemiluminescence as a measurement parameter [40], based on the same principle as Test No. 455. Agonists and antagonists act as AR ligands and can activate or inhibit the transcription of androgen-sensitive genes. The test method is used to establish signal activation or blocking of the AR caused by a ligand. Following ligand binding, the receptor–ligand complex translocates to the nucleus, where it binds specific DNA response elements and transactivates a firefly luciferase reporter gene, resulting in an increased cellular expression of the luciferase enzyme. Luciferin is a substrate that is transformed by the luciferase enzyme into a bioluminescence product that can be quantitatively measured using a luminometer.

The AR-EcoScreenTM cell line is derived from a Chinese hamster ovary cell line (CHO-K1) and includes three stably inserted chimeras.

The AR-CALUX^®^ uses the AR-CALUX cell line derived from human osteosarcoma that expresses stably transfected human AR.

The ARTA method uses a 22Rv1/MMTV_GR-KO cell line derived from a human prostate cancer cell line (22Rv1).

The purpose of these assays is to assess the transcriptional activation and inhibition of AR-mediated responses. They provide information on the concentration-response relationship of substances with agonistic or antagonistic androgenic activity in vitro. The various tests are compared in Section 6.

## 5. Impact on Hormonal Synthesis

Endocrine disruptors can interfere with hormone synthesis or transport and, in particular, target the enzymes required for steroidogenesis. Steroidogenesis is the process of the synthesis of steroid hormones from cholesterol. Different enzymes that catalyze different reactions are involved in the biosynthesis of sex steroid hormones.

**Figure 2:**
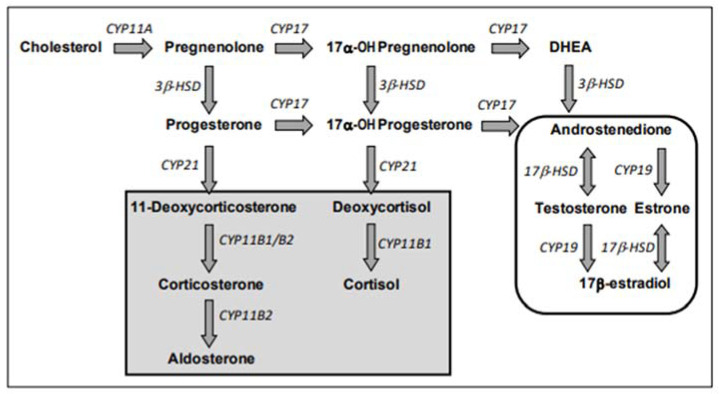
Steroidogenic pathway (from Test No. 456: H295R Steroidogenesis Assay [41]. Enzymes are in italics, hormones are bolded, and arrows indicate the direction of synthesis. Gray background indicates corticoid pathways/products. Sex steroid pathways/products are circled. CYP = cytochrome P450; HSD = hydroxysteroid hydrogenase; DHEA = dehydroepiandrosterone.

E2 and testosterone (T) are considered final hormones of the steroidogenic pathway. They are obtained via several chemical reactions, the first reaction of which is the enzymatic conversion of cholesterol to pregnenolone by the cholesterol side chain cleavage enzyme (CYP11A), related to cytochrome P450 (CYP); see Figure 2.

Steroidogenesis is divided into two pathways, the Δ5-hydroxysteroid pathway and the Δ4 -ketosteroid pathway, each leading to the production of androstenedione from pregnenolone. Androstenedione is converted by 17β-hydroxysteroid dehydrogenase (17β-HSD) to T. Then, T is converted to E2 by aromatase (CYP19). T is therefore both an intermediate and a final hormone. Androstenedione is also converted to estrone by aromatase (CYP19), which is then converted to E2.

One assay evaluating the effects of a substance on hormonal synthesis has been validated by the OECD: Test No. 456: H295R Steroidogenesis Assay. Test No. 456 is an in vitro assay to detect the effects of chemical products on steroidogenesis [41]. The H295R human adrenal carcinoma cell line is used because of its specific ability to express, on its own, the genes that encode all the key enzymes for steroidogenesis. The expression of these genes is specific to the tissue and stage of development. Typically, no tissue or stage of development expresses all the genes involved in steroidogenesis. These cells allow testing for substances that affect corticosteroid synthesis and sex steroid hormone production. The detection of substances influencing the production of testosterone and E2 is particularly studied. In this assay, cells are placed in multi-well culture plates for the time necessary for their adaptation. The cells are then exposed to the chemical test product. At the end of the exposure period, the medium is removed from the wells. Cell viability is analyzed, and the concentration of hormones in the medium is measured. This is carried out using commercial hormone assay kits or via instrumental techniques. The comparison between all OECD tests is presented in Section 6.

The OCSPP Assay 8901200: Aromatase Test detects interference of endocrine disruptors with aromatase activity. Aromatase, the enzyme responsible for the conversion of androgens to estrogens, is involved in the final step of steroidogenesis. Encoded by the CYP19 gene, aromatase converts androgens into estrogens and forms an electron transfer complex with its partner, NADPH-cytochrome P450 reductase. Upon binding to estrogen, the ER activates the transcription of its target genes, which are notably responsible for cancer cell proliferation in estrogen-dependent breast tumors [42]. Modulation of CYP19 aromatase expression and function can significantly alter the rate of estrogen production, disrupting local and systemic estrogen levels [43]. Aromatase is likely to be the target of endocrine disruptors, which may alter its expression or activity [44]. The purpose of Assay 890.1200 is to detect the interference of endocrine disruptors with aromatase activity [38]. This test is based on the use of microsomes containing recombinant human CYP19 and cytochrome P450 reductase. It measures the conversion of androgens to estrogens in microsomes isolated from various tissues or cell lines. A radioactive substrate (^3^H-androstenedione) and NADPH are added to microsomes. During the conversion of androstenedione to estrone, tritiated water (^3^H_2_O) is released and is quantitatively associated with aromatase activity. The advantages and inconveniences of all guidelines are presented in Section 6.

## 6. Advantages and Limits of the Presented International Guidelines

### 6.1. In Vitro Tests Validated by OECD

The main advantages and limits of *in vitro* tests validated by OECD are presented in Table 1.

### 6.2. In Vitro Tests Validated by the OCSPP (Office of Chemical Safety and Pollution Prevention)

The main advantages and limits of OCSPP test guidelines are presented in Table 2.

## 7. Need for New Methods

The available test guidelines mainly focus on a few endocrine pathways (i.e., estrogenic, androgenic, as well as steroidogenesis), leaving many unexplored, such as the retinoid or glucocorticoid pathways.

Given the numerous limitations of available international guidelines, some platforms have been created to organize the pre-validation of methods for characterizing endocrine disruptors. The first platform is the public–private PEPPER platform (public–private platform for the pre-validation of testing methods on endocrine disruptors), created in 2019 [45]. The project has been piloted by stakeholders from both industrial sectors and ministerial divisions. It is part of the National Strategy on Endocrine Disruptors (SNPE), designed by the French ministries in charge of Health and in charge of the Environment [46,47]. The platform ensures that candidate methods are sufficiently mature to enter pre-validation and are of regulatory relevance to be presented to international bodies responsible for validation, such as OECD, ECVAM, ISO, and CEN [48]. Once these methods are validated by the relevant bodies, they can be used in regulatory dossiers related to substances (e.g., REACH, Biocides, and Phytopharmaceuticals), products (e.g., medical devices), or environments (e.g., Water Framework Directive).

For pre-validation, a developing laboratory and a minimum of two testing laboratories must participate in the process. The first phase, called transferability, involves ensuring that a laboratory that is not familiar with the method can successfully implement it and obtain results consistent with those obtained by the laboratory that developed it by following the procedures provided. This is carried out by applying the method to a small set of substances, chosen based on the developer’s “historical data”, and determined by the Validation Management Group (VMG), composed of experts on the subject. In the second phase, all laboratories blind test a larger number of test substances, also defined with the VMG, allowing for a better understanding of the method’s applicability domain.

The present review focuses on PEPPER pre-validated methods as it is the first platform created. To date, nine methods have been selected by PEPPER for validation. Three of them have been successfully transferred to (2–3) naïve labs: the h-Placentox method, the Glucocorticoid Receptor Transactivation Assay (GR TA), and the Retinoid Acid Receptor TransActivation method. Three are in the transferability process: the LCMS steroidogenesis profiling method, the sexual development of the embryo in hen eggs, and the hNPC proliferation arrest method. Transferability has started for two others: the in vitro assay for hepatic triglyceride accumulation and the Mineralocorticoid Receptor Transactivation Assay. The last one is just being launched: the Deiodinase 1 (DIO1) activity.

Their further assessment is ongoing by testing 12 to 35 substances (depending on the method). Five methods have been included in the OECD workplan: the LCMS steroidogenesis profiling method, the h-Placentox method, the Glucocorticoid Receptor Transactivation Assay (GR TA) in April 2022, and the Retinoid Acid Receptor TransActivation method and the sexual development of avian embryo in April 2023. They are based on novel endpoints that could provide new, in-depth information to assess chemicals for their endocrine disruptor potential. We note that the methods that follow the validation process will potentially be modified at the end of the process.

### 7.1. The LCMS Steroidogenesis Profiling Method

The LCMS steroidogenesis profiling method studies the effects of substances on hormone synthesis using human adrenal cells and is an extension of Test Guideline No. 456: H295R Steroidogenesis Assay Guideline [41]. The method is meant to simultaneously analyze 19 steroids, representing the steroid biosynthesis pathway. Increasing the number of parameters measured in TG 456 beyond E2 and T would maximize its regulatory utility by providing more information on the disruption of steroidogenesis via the identification of endocrine modes of action beyond estrogens and androgens. This method has been developed by the Research Institutes of Norway [49].

The choice of LCMS/MS measurement type is related to the high number of parameters to be measured, which would be difficult to monitor by the immuno-enzymatic method. ELISA (Enzyme-Linked Immunosorbent Assay) and RIA (Radioimmunoassay) can only measure 1 hormone at a time and may be influenced by cross-reactivity, whereas chromatographic techniques allow for the simultaneous detection of several steroids in the pathway without any cross-reactivity. However, this type of equipment (LCMS) is not available in all laboratories or yet mastered for those parameters.

Another advantage of the method is that the H295R cell line is available in international cell banks and then easy to obtain.

The limits are the same as those identified with TG 456:

- Long doubling time for the H295R cell line;
- Restricted number of cell passages;
- Unknown metabolic capacity of the cell line;
- Does not determine which enzymes are affected by the chemical substance due to the presence of all enzymes involved in steroidogenesis in this cell line;
- Does not identify substances that interfere with steroidogenesis due to effects on the hypothalamic–pituitary–gonadal (HHG) axis.

Furthermore, increasing the number of measured parameters will require expanding the data interpretation procedure and the prediction model. To improve the limit of detection of the 19 precursors, dilution and/or concentrations of the sample may be necessary, which considerably weighs down the process.

### 7.2. The h-Placentox Method

The h-Placentox method is the second method to enter the process. It uses human placental cells to evaluate the effect of maternal exposure during pregnancy.

The placenta allows the growth and development of the fetus by coordinating gas exchange, metabolic transfer, and immunological functions and by producing, metabolizing, and regulating numerous hormones, notably polypeptide and steroid hormones.

There are several different in vitro approaches currently available to evaluate chemical toxicity in the human placenta. The human placental JEG-3 cell line is close to human physiology and appears to be the best tool for the assessment of chemical toxicity in the placenta and is therefore used for this test [50].

The JEG 3 cell line secretes both polypeptide and steroid hormones and expresses the functional P2X7 cell degeneration membrane receptor. The P2X7 receptor is a ubiquitous membrane receptor that induces many intracellular signaling pathways after alterations of the ion permeability or after the formation of a large pore, depending on the duration of the stimulus. Pore formation after prolonged activation of the P2X7 receptor leads to apoptosis. P2X7 receptor activation is reported to be involved in multiple pathologies, from immune disorders to degenerative diseases [51,52,53,54,55,56]. There is evidence to suggest that the P2X7 receptor may play a role in the pathophysiology of various placental disorders, including preeclampsia and intrauterine growth restriction (IUGR) [57]. It has been shown that targeting the P2X7 receptor specifically could offer an effective approach to prevent both preterm birth and perinatal brain injury associated with exposure to intrauterine inflammation [58,59].

The h-Placentox test is based on the activation of the P2X7 receptor as an in vitro marker of adverse health effects and the dosage of hormones. It has been used to evaluate forskolin, a diterpene from a plant used in food supplements for weight loss [60], essential oils [61], and chlorpyrifos, a persistent and bioaccumulative worldwide banned pesticide [62].

The analysis techniques are multiple, and among them, we find the analysis of the activation of the P2X7 receptor by means of the YO-PRO1 probe. This is a fluorogenic probe that can only enter the cell after activation of P2X7 receptors [63]. Once inside, it binds to nucleic acids and emits fluorescence. However, this probe is only available from one supplier, which could be a problem if the probe is discontinued.

The main advantage of this method is that, contrary to the other methods (validated and under validation), it evaluates both the alteration of hormone secretion and the induction of a receptor involved in placental pathologies. In addition, it allows the screening of many molecules. Plus, the JEG-3 cell line is available in international cell banks like the American Type Culture Collection ATCC.

Culture conditions may affect the sensitivity of the cells to endocrine disruptors, so attention should be paid mainly to the culture medium content and standardization of the bovine fetal serum as it is rich in hormones [64]. Plus, the not-fully described metabolic capacity of the JEG-3 cell line may represent a limit of the method.

### 7.3. The Glucocorticoid Receptor Transactivation Assay (GR TA)

The GR TA measures the agonistic/antagonistic effect of a substance on the human glucocorticoid receptor via luciferase activity. This test uses transfected HeLa human cells obtained from cervical adenocarcinoma. This test requires the co-transfection of the HeLa cell line with a glucocorticod-responsive gene, MMTVLuc-SV-Neo, and a glucocorticoid receptor expressing plasmid.

The human glucocorticoid receptor (hGR) is involved in several physiological processes, such as stress response, immune system, and metabolism [65,66,67]. Because of the involvement of this receptor in physiological processes of primary importance, it is important to evaluate the ability of chemicals to interfere with this nuclear receptor.

The expression of hGR is ubiquitous, and its ligands include endogenous and synthetic glucocorticoids such as cortisol, dexamethasone, and prednisolone [68].

In one study, the authors of this method tested the effect of bisphenol A and 24 analogs for their ability to modulate GR activity [69]. They demonstrated that nuclear receptors were differentially affected by bisphenols. The activity of the nuclear receptors is either activated or antagonized by bisphenols. In this paper, the glucocorticoid receptor bound a smaller number of bisphenols and was always antagonized by bisphenols.

The assay has also been proposed to measure endocrine activity in drinking water [70].

### 7.4. Sexual Development of Avian Embryo

The method, which studies the sexual development of the avian embryo, was developed by Uppsala University. The principle of the method relies on the exposition of chicken embryos to chemicals followed by the observation of reproductive organs and aromatase gene expression [71]. Here, the chicken embryo is used as a whole model; it involves studying and manipulating the living embryo itself, which makes it an in vitro model, even if not covered by the Directive on the protection of animals used for scientific purposes (Dir 2010/63/EU) due to the early stage of development considered.

This method, when submitted to the OECD work plan, has been coupled with a similar method from Japan on quail embryos under the name of “avian in ovo assay”. These methods have been welcomed by OECD members, as very few methods are currently available for birds.

### 7.5. The In Vitro RAR TA (Retinoic Acid Receptor TransActivation) Method

The retinoid system is involved in many functions, such as reproduction, differentiation, development, and sight [72,73,74]. The effect of retinoids is mediated via nuclear receptors, RAR (alpha, beta, and gamma) and RXR (alpha, beta, and gamma) [75]. In 2012, the OECD Test Guidelines Programme in a Detailed Review Paper (DRP 178) identified a need for harmonized test methods for the retinoid system for toxicity screening and evaluation. Sweden and the European Commission initiated work on a DRP to review knowledge on the retinoid signaling pathway in multiple organ systems, which was subsequently narrowed to four areas: Overview, Reproductive System, Skeletal Patterning, and Central Nervous System Development [25,76]. Retinoic Acid Receptor transactivation methods were highlighted as of interest for regulatory purpose [77].

The method is based on a luciferase reporter cell line first incubated with the product to be tested, and then with luciferin to measure the transactivation of the RAR [78,79]. The method allows for the discrimination between RAR and RXR activation.

### 7.6. The In Vitro Human Neural Progenitor Cell (hNPC) Proliferation Arrest Method

Research based on data from birth cohorts has revealed that when individuals are exposed to endocrine disruptors during crucial stages of neurodevelopment, it can have detrimental effects on various cognitive functions, such as memory and language, as well as attention, emotions, and social behaviors [80,81]. Immortalized human neural progenitor cells (hNPCs) cultivated as three-dimensional floating spheres can represent processes of brain development [82,83]. After exposition to potential endocrine disruptors, the process of NPC proliferation can be studied [84,85]. The method under validation relies on this concept and was developed by the German Leibniz Research Institute for Environmental Medicine (IUF) [86,87,88]. The method focuses on proliferation (stimulation or inhibition) mediated by the retinoid or glucocorticoid pathways. This method could be used in the currently developed in vitro battery of tests to study developmental neurotoxicity.

### 7.7. In Vitro Assay for Hepatic Triglyceride Accumulation

Liver steatosis refers to the buildup of fat in the liver, which can potentially lead to non-alcoholic fatty liver disease (NAFLD) [89,90,91]. In severe cases, it may progress to liver fibrosis, causing significant damage. NAFLD is recognized as the most prevalent liver disorder worldwide, affecting approximately 32% of the global population [92]. The crucial role of environmental pollutants, particularly endocrine disruptors, in the development of NAFLD has been acknowledged [93,94].

To assess hepatic triglyceride accumulation, an in vitro assay has been developed by the German Federal Institute for Risk Assessment (BfR). This quantitative test method specifically examines the intracellular accumulation of triglycerides in HepaRG liver cells, a widely utilized model for studying human hepatocytes. The accumulation is detected using a fluorescent dye, like the AdipoRed reagent (Lonza, Basel, Switzerland) described by Lasch et al. [95]. This method evaluates metabolic disorders, an area recognized as lacking methods for regulatory assessment.

### 7.8. Deiodinase 1 (DIO1) Activity Based on Sandell–Kolthoff (SK) Reaction

Thyroid hormones serve as crucial regulators of growth, development, and overall homeostasis in vertebrates [96,97,98]. The process of deiodination, facilitated by the enzyme deiodinase (DIO1), plays a fundamental role in regulating the concentration of thyroid hormones in peripheral tissues and plasma [99,100]. Although there are numerous contaminants known to disrupt the action of thyroid hormones [101,102,103], the mechanisms behind these disruptions are only partially understood, resulting in a gap in current methods for detecting such chemicals.

It has been demonstrated that certain chemicals can interfere with the activity of DIO1, consequently affecting thyroid function [104].

The DIO1 activity based on Sandell–Kolthoff (SK) reaction uses a colorimetric Sandell–Kolthoff (SK) reaction to quantify DIO1 activity in cell material. This assay has been developed by the German Federal Institute for Risk Assessment (BfR) and Charité-Universitätsmedizin Berlin. The transferability of this test to a naïve lab has been evaluated as part of the work initiated by EURL-ECVAM with the EU-Netval network [105].

### 7.9. Mineralocorticoid Receptor Transactivation Assay (MR TA)

Glucocorticoid receptors (GRs) and mineralocorticoid receptors (MRs) mediate the actions of glucocorticoids and mineralocorticoids, respectively, which are two main classes of corticosteroids involved in many physiological processes. The GRs and the MRs are potential targets for endocrine disruptors, which interfere with GR/MR activity by disrupting ligand/DNA–receptor binding, GRs/MRs expression, and translocation [106].

The under-validation test measures the activation/inhibition of the human MR via a luciferase reporter gene. It is based on the human osteosarcoma cells (U2OS human cells) stably expressing the ligand binding domain of human MR fused to the yeast GAL4 DNA binding domain. It is a stable model that allows specific and sensitive measurement of the activities of the ligands of the MR. In addition, it provides a high-throughput cellular screening tool for the identification and characterization of MR ligands, either agonists or antagonists.

### 7.10. Comparison to Existing OECD and OCSPP Guidelines

These new methods have the potential to address the gaps left by the OECD and OCSPP guidelines. However, certain limitations persist, including limited or unknown metabolic capabilities of the cell model and a lack of information regarding the test’s applicability to mixtures. Metabolism can significantly affect the toxicity and endocrine-disrupting potential of chemicals; therefore, preliminary studies should be performed to prove the ability of cellular models used in endocrine disruption risk assessment to metabolize the assessed chemical. For example, assaying the activity of specific enzymes, such as cytochrome P450s (CYPs) or phase II conjugation enzymes, can provide insights into the metabolic capacity of a cell line. Examining the gene expression profile encoding for specific metabolic enzymes can also give indications of the cell line’s metabolic capabilities.

When evaluating mixtures, a case-by-case approach should be used. Indeed, mixtures can have varying compositions, interactions, and potential effects, and therefore, they may require different methods or considerations in the evaluation process.

Additionally, the WHO definition of endocrine disruptors mentions “the adverse health effects in an intact organism, or its progeny, or (sub)populations”. In vitro models often isolate individual tissues or cell types, making it challenging to study tissue-tissue interactions and their role in endocrine disruption. Co-culture in 3D models is an approach that involves the cultivation of multiple types of cells in a three-dimensional environment to mimic the complex interactions that occur within living organisms more closely than traditional 2D cell cultures. These models would provide a more physiologically relevant platform for studying cell–cell interactions, cell signaling pathways, and tissue development. Nevertheless, co-culture in 3D models comes with challenges, including the need for complex culture systems and difficulties in quantifying cell interactions. Particular attention must be paid to the choice of cell types, their ratios, and the culture conditions to replicate specific biological processes accurately.

## 8. Discussion and Conclusions

Efforts are being made on a global scale to improve the evaluation and understanding of the endocrine-disrupting potential of chemicals. The recognition of the impact that endocrine disruptors can have on human health and the environment has led to increased attention and research in this field. These efforts involve various stakeholders, including scientists, regulatory agencies, policymakers, and industry representatives, who collaborate to develop robust methodologies and guidelines for assessing endocrine disruptors. One key aspect of these initiatives is the development of standardized testing protocols and guidelines. These guidelines aim to provide consistent and reliable methods for identifying and characterizing endocrine disruptors. They outline specific endpoints, study designs, and evaluation criteria to ensure that the potential endocrine-disrupting properties of chemicals are thoroughly evaluated. Before entering the cycle of validation, these tests must be mature enough. Recently, an online self-assessment questionnaire (SAQ) called ReadEDTest has been developed to speed up the validation process by assessing readiness criteria of in vitro and fish embryo endocrine disruptors test methods under development [107]. The SAQ is divided into 7 sections and 13 sub-sections containing essential information requested by the validating bodies. According to the authors, ReadEDTest provides test developers who may not be familiar with international validation requirements with a straightforward and efficient way to obtain answers about crucial aspects of their test methods. This enables them to easily identify the strengths and limitations of their methods, gauging their readiness for potential validation.

Regardless of the biological issue being studied, from genotoxicity, which is a simple phenomenon studying the interaction of a substance with DNA, to much more complex phenomena, such as endocrine disruption, it is impossible to draw conclusions based on a single assay. In the safety assessment of cosmetic ingredients, the SCCS 10th Revision recommended to use of an in vitro battery of two tests to evaluate genotoxicity [108]. One in vitro test for the evaluation of the potential for mutagenicity and a second in vitro micronucleus test for the evaluation of chromosome damage (clastogen and aneuploidy). The combination of both tests allowed the detection of all relevant genotoxic carcinogens [109,110]. When the genotoxicity of a medical device has to be experimentally assessed, a series of in vitro tests shall be used, and at least two tests investigating different end-points shall use mammalian cells [111]. A similar approach should be applied to endocrine disruptors. When evaluating the potential endocrine-disrupting activity of chemicals, the evaluators must rely on the weight of evidence approach to consider various types of data from multiple sources. The weight of evidence approach allows the evaluators to assess the overall strength, consistency, and reliability of the data to determine the likelihood of a substance being an endocrine disruptor. The weight of evidence approach considers not only the quantity of evidence but also its quality and relevance. The guidance for the identification of endocrine disruptors in the context of Regulations (EU) N°528/2012 and (EC) No 1107/2009 aims to assist applicants and assessors from competent regulatory authorities in identifying endocrine disruptors [28]. This guidance outlines the process of gathering, evaluating, and considering all relevant information during the assessment of potential endocrine disruptors. It also explains how to conduct a mode of action analysis and utilize a weight of evidence approach to determine whether the endocrine disruptors criteria are met.

The strength of OECD guidelines lies in their scientific rigor, transparency, and consensus-based development process. These guidelines are developed by expert scientists and representatives from OECD member countries, ensuring a broad range of expertise and perspectives are considered. The above-described methods under validation by the OECD do not fully overcome the limitations of validated international guidelines. For example, the metabolic capacity of cells remains a potentially problematic aspect. Metabolic capacity is a crucial aspect of toxicology. If the metabolic capacity of cells is not adequately considered or accounted for, it can lead to inaccurate or incomplete results, affecting the overall reliability and validity of the research or interventions. Therefore, although developmental methods may offer advancements in risk assessment related to endocrine disruptors, they still fall short in addressing the limitations of validated guidelines, particularly in relation to the metabolic capacity of cells.

The test battery approach is designed to reduce the risk of false negative results for compounds with toxic potential. It is important to keep exploring and developing new methodologies that can effectively evaluate the risks associated with potential endocrine disruptors. By integrating these methods into a comprehensive weight-of-evidence framework, we can enhance our understanding of the potential hazards and make informed decisions regarding the regulation and management of these substances. This ongoing pursuit will contribute to more robust and reliable assessments, ensuring the protection of human health and the environment from the adverse effects of endocrine disruptors.

## Figures and Tables

**Table 1:** Advantages and limits of OECD test guidelines.

Test	Advantages	Limits
**Transcriptional activation/inhibition assays**		
Test No. 455: Performance-Based Test Guideline for Stably Transfected Transactivation In vitro Assays to Detect Estrogen Receptor Agonists and Antagonists	HeLa9903 cell line of human origin available in international cell banks, easy to obtain. VM7Luc4E2 cell line of human origin but available under a technical license agreement from the University of California at Davis (CA, USA) and from Xenobiotic Detection Systems Inc. in Durham (NC, USA), quite easy to obtain. Calux cell line of human origin but available under a technical license agreement from Bio Detection Systems in Amsterdam (The Netherlands), quite easy to obtain. Both agonist and antagonist activity (estrogen). Considers the potential impact of the tested chemical product on cell viability. Key mechanisms of ER-mediated endocrine disruption.	Focuses only on the transcriptional activation or inhibition of an ER-regulated reporter gene. Limited metabolic capabilities. Cannot be extrapolated to estrogen signaling and regulation. Risk of false positives with phytoestrogens. No information on the applicability of the test to mixtures.
Test No. 458: Stably Transfected Human Androgen Receptor Transcriptional Activation Assay for Detection of Androgenic Agonist and Antagonist Activity of Chemicals	AR-EcoScreenTM cell line available in international cell banks, easy to obtain. Calux cell line of human origin but available under a technical license agreement from Bio Detection Systems in Amsterdam (The Netherlands). 22Rv1 cell line of human origin available in international cell banks, easy to obtain. Both agonist and antagonist activity (androgen). Considers the potential impact of the tested chemical product on cell viability. Key mechanisms of AR-mediated endocrine disruption.	AR-EcoScreenTM cell line of animal origin. Limited metabolic capabilities. Focuses only on the AR. Risk of crosstalk interference with GR if the chosen cell line expresses the glucocorticoid receptor. No information on the applicability of the test to mixtures.
**Interaction tests of a substance on hormonal synthesis**		
Test No. 456: H295R Steroidogenesis Assay	H295R cell line available in international cell banks, easy to obtain. Can determine both increases and inhibitions of T and E2 (steroid) hormones secretion. Considers the potential impact of the tested chemical product on cell viability. H295R cells share physiological characteristics of zonally undifferentiated human fetal adrenal cells. They can produce all steroid hormones found in the adult adrenal cortex and gonads.	Unknown metabolic capacity of the cell line. Does not determine which enzyme is affected by the chemical substance due to the presence of all enzymes involved in steroidogenesis in this cell line. Does not identify substances that interfere with steroidogenesis due to effects on the hypothalamic-pituitary-gonadal (HHG) axis. Long doubling time for the H295R cell line. Restricted number of cell passages. No information on the applicability of the test to mixtures.
**Binding tests of a substance to a receptor**		
Test No. 493: Performance-Based Test Guideline for Human Recombinant Estrogen Receptor (hrER) In vitro Assays to Detect Chemicals with ER Binding Affinity	Inexpensive. Key mechanisms of ER-mediated endocrine disruption. High throughput screening.	Does not consider other mechanisms, such as interactions with parts of ERα other than the ligand binding site and interactions with other receptors involved in estrogen signaling. Does not distinguish between ERα agonists and antagonists. No information on the applicability of the test to mixtures. Not applicable to chemical products that may denature proteins (such as surfactants). The use of a radiolabeled ligand requires authorization to handle radioactive materials.

**Table 2:** Advantages and limits of OCSPP test guidelines.

Test	Advantages	Limits
**Binding tests of a substance to a receptor**		
Assay 890.1150: Androgen receptor binding assay	Inexpensive. Key mechanisms of AR-mediated endocrine disruption. High throughput screening.	In chemico test (no living material). Derived from animal (rat) prostate. Does not distinguish between AR agonists and antagonists. The use of a radiolabeled ligand requires authorization to handle radioactive materials. No information on the applicability of the test to mixtures.
**Interaction tests of a substance on hormonal synthesis**		
Assay 890.1200: Aromatase Test	Inexpensive. Provides an early indication of potential endocrine disruption caused by chemicals, as aromatase activity is one of the earliest events in estrogen biosynthesis. Using the cytochrome P450 reductase, which is one of the most studied endocrine disruptors studies. In addition, recombinant human microsomes containing cytochrome P450 reductase are commercially available. High throughput screening.	In chemico test (no living material). The use of a radiolabeled ligand requires authorization to handle radioactive materials. No information on the applicability of the test to mixtures.

## Data Availability

No new data were created or analyzed in this study. Data sharing is not applicable to this article.

## References

[1] WHO/IPCS (WHO, International Programme on Chemical Safety) (2002). Global Assessment of the State-of-the-Science of Endocrine Disruptors. WHO/PCS/EDC/02.2. https://www.who.int/publications/i/item/WHO-PSC-EDC-02.2.

[2] Kabir E.R., Rahman M.S., Rahman I. (2015). A Review on Endocrine Disruptors and Their Possible Impacts on Human Health. Environ. Toxicol. Pharmacol..

[3] Salazar P., Villaseca P., Cisternas P., Inestrosa N.C. (2021). Neurodevelopmental Impact of the Offspring by Thyroid Hormone System-Disrupting Environmental Chemicals during Pregnancy. Environ. Res..

[4] Lahimer M., Abou Diwan M., Montjean D., Cabry R., Bach V., Ajina M., Ben Ali H., Benkhalifa M., Khorsi-Cauet H. (2023). Endocrine Disrupting Chemicals and Male Fertility: From Physiological to Molecular Effects. Front. Public Health.

[5] Raja G.L., Subhashree K.D., Kantayya K.E. (2022). In Utero Exposure to Endocrine Disruptors and Developmental Neurotoxicity: Implications for Behavioural and Neurological Disorders in Adult Life. Environ. Res..

[6] He J., Xu J., Zheng M., Pan K., Yang L., Ma L., Wang C., Yu J. (2024). Thyroid Dysfunction Caused by Exposure to Environmental Endocrine Disruptors and the Underlying Mechanism: A Review. Chem.-Biol. Interact..

[7] Syed S., Qasim S., Ejaz M., Sammar, Khan N., Ali H., Zaker H., Hatzidaki E., Mamoulakis C., Tsatsakis A. (2023). Effects of Dichlorodiphenyltrichloroethane on the Female Reproductive Tract Leading to Infertility and Cancer: Systematic Search and Review. Toxics.

[8] European Commission (2022). Delegated Regulation Amending Regulation 1272/2008 as Regards Hazard Classes and Criteria for the Classification, Labelling and Packaging of Substances and Mixtures. https://environment.ec.europa.eu/publications/clp-delegated-act_en.

[9] (2022). Official Journal of the European Union Commission Delegated Regulation (EU) 2023/707 Introduces New Hazard Classes and Criteria for the Classification, Labelling and Packaging of Substances and Mixtures 2023.

[10] Liu A., Seal S., Yang H., Bender A. (2023). Using Chemical and Biological Data to Predict Drug Toxicity. SLAS Discov..

[11] Szczęsna D., Wieczorek K., Jurewicz J. (2022). An Exposure to Endocrine Active Persistent Pollutants and Endometriosis—A Review of Current Epidemiological Studies. Environ. Sci. Pollut. Res..

[12] Combarnous Y., Nguyen T.M.D. (2022). Membrane Hormone Receptors and Their Signaling Pathways as Targets for Endocrine Disruptors. J. Xenobiot..

[13] Varticovski L., Stavreva D.A., McGowan A., Raziuddin R., Hager G.L. (2022). Endocrine Disruptors of Sex Hormone Activities. Mol. Cell. Endocrinol..

[14] Amir S., Shah S.T.A., Mamoulakis C., Docea A.O., Kalantzi O.-I., Zachariou A., Calina D., Carvalho F., Sofikitis N., Makrigiannakis A. (2021). Endocrine Disruptors Acting on Estrogen and Androgen Pathways Cause Reproductive Disorders through Multiple Mechanisms: A Review. Int. J. Environ. Res. Public Health.

[15] Liang Y., Gong Y., Jiang Q., Yu Y., Zhang J. (2023). Environmental Endocrine Disruptors and Pregnane X Receptor Action: A Review. Food Chem. Toxicol..

[16] Tabęcka-Łonczyńska A., Kaczka P., Kaleniuk E. (2023). Involvement of Estrogen Receptor Alpha (ERα) and Impairment of Steroidogenesis after Exposure to Tris(2,3-Dibromopropyl) Isocyanurate (TBC) in Mouse Spermatogenic (GC-1 Spg) Cells in Vitro. J. Steroid Biochem. Mol. Biol..

[17] Endocrine Society (2024). Endocrine-Related Organs and Hormones.

[18] Usta S.N., Scharer C.D., Xu J., Frey T.K., Nash R.J. (2014). Chemically Defined Serum-Free and Xeno-Free Media for Multiple Cell Lineages. Ann. Transl. Med..

[19] Czapla J., Matuszczak S., Kulik K., Wiśniewska E., Pilny E., Jarosz-Biej M., Smolarczyk R., Sirek T., Zembala M.O., Zembala M. (2019). The Effect of Culture Media on Large-Scale Expansion and Characteristic of Adipose Tissue-Derived Mesenchymal Stromal Cells. Stem. Cell Res. Ther..

[20] Vandenberg L.N., Colborn T., Hayes T.B., Heindel J.J., Jacobs D.R., Lee D.-H., Shioda T., Soto A.M., vom Saal F.S., Welshons W.V. (2012). Hormones and Endocrine-Disrupting Chemicals: Low-Dose Effects and Nonmonotonic Dose Responses. Endocr. Rev..

[21] Autrup H., Barile F.A., Berry S.C., Blaauboer B.J., Boobis A., Bolt H., Borgert C.J., Dekant W., Dietrich D., Domingo J.L. (2020). Human Exposure to Synthetic Endocrine Disrupting Chemicals (S-EDCs) Is Generally Negligible as Compared to Natural Compounds with Higher or Comparable Endocrine Activity. How to Evaluate the Risk of the S-EDCs?. Environ. Toxicol. Pharmacol..

[22] Zhang X., Wu C. (2022). In Silico, In Vitro, and In Vivo Evaluation of the Developmental Toxicity, Estrogenic Activity, and Mutagenicity of Four Natural Phenolic Flavonoids at Low Exposure Levels. ACS Omega.

[23] European Commission EU Science Hub Interlaboratory Comparisons.

[24] (1999). Chapter 12 Interlaboratory Studies. Techniques and Instrumentation in Analytical Chemistry.

[25] OECD (2018). Revised Guidance Document 150 on Standardised Test Guidelines for Evaluating Chemicals for Endocrine Disruption.

[26] (2012). Official Journal of the European Union Regulation (EU) No 528/2012 of the European Parliament and of the Council of 22 May 2012 Concerning the Making Available on the Market and Use of Biocidal Products 2012.

[27] (2009). Regulation (EC) No 1107/2009 of the European Parliament and of the Council of 21 October 2009 Concerning the Placing of Plant Protection Products on the Market and Repealing Council Directives 79/117/EEC and 91/414/EEC 2009.

[28] Andersson N., Arena M., Auteri D., Barmaz S., Grignard E., Kienzler A., Lepper P., Lostia A.M., Munn S., European Chemical Agency (ECHA) and European Food Safety Authority (EFSA) with the technical support of the Joint Research Centre (JRC) (2018). Guidance for the Identification of Endocrine Disruptors in the Context of Regulations (EU) No 528/2012 and (EC) No 1107/2009. EFS2.

[29] La Merrill M.A., Vandenberg L.N., Smith M.T., Goodson W., Browne P., Patisaul H.B., Guyton K.Z., Kortenkamp A., Cogliano V.J., Woodruff T.J. (2020). Consensus on the Key Characteristics of Endocrine-Disrupting Chemicals as a Basis for Hazard Identification. Nat. Rev. Endocrinol..

[30] Endocrine Society Endocrine Society Endocrine-Disrupting Chemicals (EDCs) 2022.

[31] Guarnotta V., Amodei R., Frasca F., Aversa A., Giordano C. (2022). Impact of Chemical Endocrine Disruptors and Hormone Modulators on the Endocrine System. Int. J. Mol. Sci..

[32] Ahn C., Jeung E.-B. (2023). Endocrine-Disrupting Chemicals and Disease Endpoints. Int. J. Mol. Sci..

[33] Endocrine Society Impact of EDCs on Hormone-Sensitive Cancer.

[34] Gaspari L., Soyer-Gobillard M.-O., Kerlin S., Paris F., Sultan C. (2024). Early Female Transgender Identity after Prenatal Exposure to Diethylstilbestrol: Report from a French National Diethylstilbestrol (DES) Cohort. J. Xenobiot..

[35] Soyer-Gobillard M.-O., Gaspari L., Courtet P., Sultan C. (2022). Diethylstilbestrol and Autism. Front. Endocrinol..

[36] Tabb M.M., Blumberg B. (2006). New Modes of Action for Endocrine-Disrupting Chemicals. Mol. Endocrinol..

[37] OECD (2015). Test No. 493: Performance-Based Test Guideline for Human Recombinant Estrogen Receptor (hrER) In Vitro Assays to Detect Chemicals with ER Binding Affinity.

[38] United States Environmental Protection Agency (EPA) (2009). Endocrine Disruptor Screening Program Test Guidelines—OPPTS 890.1150 Androgen Receptor Binding (Rat Prostate Cytosol).

[39] OECD (2021). Test No. 455: Performance-Based Test Guideline for Stably Transfected Transactivation In Vitro Assays to Detect Estrogen Receptor Agonists and Antagonists.

[40] OECD (2023). Test No. 458: Stably Transfected Human Androgen Receptor Transcriptional Activation Assay for Detection of Androgenic Agonist and Antagonist Activity of Chemicals.

[41] OECD (2023). Test No. 456: H295R Steroidogenesis Assay.

[42] Hong Y., Li H., Yuan Y.-C., Chen S. (2009). Molecular Characterization of Aromatase. Ann. N. Y. Acad. Sci..

[43] Cheshenko K., Pakdel F., Segner H., Kah O., Eggen R.I.L. (2008). Interference of Endocrine Disrupting Chemicals with Aromatase CYP19 Expression or Activity, and Consequences for Reproduction of Teleost Fish. Gen. Comp. Endocrinol..

[44] Baravalle R., Ciaramella A., Baj F., Di Nardo G., Gilardi G. (2018). Identification of Endocrine Disrupting Chemicals Acting on Human Aromatase. Biochim. Biophys. Acta (BBA)-Proteins Proteom..

[45] French National Institute for Industrial Environment and Risks (Ineris) (2019). The Birth of the PEPPER Platform 2019.

[46] WHO (2019). French Ministries in Charge of Health and in Charge on the Environment Second National Strategy on Endocrine Disruptors. 2019–2022: Strategic Objectives 2019.

[47] WHO (2019). French Ministries in Charge of Health and in Charge on the Environment Second National Strategy on Endocrine Disruptors. 2019–2022: Action Plan. 2019.

[48] Zgheib E., Kim M.J., Jornod F., Bernal K., Tomkiewicz C., Bortoli S., Coumoul X., Barouki R., De Jesus K., Grignard E. (2021). Identification of Non-Validated Endocrine Disrupting Chemical Characterization Methods by Screening of the Literature Using Artificial Intelligence and by Database Exploration. Environ. Int..

[49] Ahmed K.E.M., Frøysa H.G., Karlsen O.A., Sagen J.V., Mellgren G., Verhaegen S., Ropstad E., Goksøyr A., Kellmann R. (2018). LC-MS/MS Based Profiling and Dynamic Modelling of the Steroidogenesis Pathway in Adrenocarcinoma H295R Cells. Toxicol. Vitr..

[50] Olivier E., Wakx A., Fouyet S., Dutot M., Rat P. (2021). JEG-3 Placental Cells in Toxicology Studies: A Promising Tool to Reveal Pregnancy Disorders. Anat. Cell Biol..

[51] Zheng H., Liu Q., Zhou S., Luo H., Zhang W. (2024). Role and Therapeutic Targets of P2X7 Receptors in Neurodegenerative Diseases. Front. Immunol..

[52] Soni S., Lukhey M.S., Thawkar B.S., Chintamaneni M., Kaur G., Joshi H., Ramniwas S., Tuli H.S. (2024). A Current Review on P2X7 Receptor Antagonist Patents in the Treatment of Neuroinflammatory Disorders: A Patent Review on Antagonists. Naunyn-Schmiedeberg’s Arch. Pharmacol..

[53] Ronning K.E., Déchelle-Marquet P.-A., Che Y., Guillonneau X., Sennlaub F., Delarasse C. (2023). The P2X7 Receptor, a Multifaceted Receptor in Alzheimer’s Disease. Int. J. Mol. Sci..

[54] Lécuyer D., Nardacci R., Tannous D., Gutierrez-Mateyron E., Deva Nathan A., Subra F., Di Primio C., Quaranta P., Petit V., Richetta C. (2023). The Purinergic Receptor P2X7 and the NLRP3 Inflammasome Are Druggable Host Factors Required for SARS-CoV-2 Infection. Front. Immunol..

[55] Zhang R., Su K., Yang L., Tang M., Zhao M., Ye N., Cai X., Jiang X., Li N., Peng J. (2023). Design, Synthesis, and Biological Evaluation of Novel P2X7 Receptor Antagonists for the Treatment of Septic Acute Kidney Injury. J. Med. Chem..

[56] Di Virgilio F., Vultaggio-Poma V., Falzoni S., Giuliani A.L. (2023). The Coming of Age of the P2X7 Receptor in Diagnostic Medicine. Int. J. Mol. Sci..

[57] Roberts V.H.J., Greenwood S.L., Elliott A.C., Sibley C.P., Waters L.H. (2006). Purinergic Receptors in Human Placenta: Evidence for Functionally Active P2X4, P2X7, P2Y2, and P2Y6. Am. J. Physiol. Integr. Comp. Physiol..

[58] Tsimis M.E., Lei J., Rosenzweig J.M., Arif H., Shabi Y., Alshehri W., Talbot C.C., Baig-Ward K.M., Segars J., Graham E.M. (2017). P2X7 Receptor Blockade Prevents Preterm Birth and Perinatal Brain Injury in a Mouse Model of Intrauterine Inflammation. Biol. Reprod..

[59] Zucker E., Burd I. (2022). P2X7 Receptor as a Potential Therapeutic Target for Perinatal Brain Injury Associated with Preterm Birth. Exp. Neurol..

[60] Rat P., Leproux P., Fouyet S., Olivier E. (2022). Forskolin Induces Endocrine Disturbance in Human JEG-3 Placental Cells. Toxics.

[61] Fouyet S., Olivier E., Leproux P., Dutot M., Rat P. (2022). Evaluation of Placental Toxicity of Five Essential Oils and Their Potential Endocrine-Disrupting Effects. Curr. Issues Mol. Biol..

[62] Fouyet S., Olivier E., Leproux P., Boutefnouchet S., Dutot M., Rat P. (2022). Cocktail Effect of Endocrine Disrupting Chemicals: Application to Chlorpyrifos in Lavender Essential Oils. Int. J. Environ. Res. Public Health.

[63] Rat P., Olivier E., Tanter C., Wakx A., Dutot M. (2017). A Fast and Reproducible Cell- and 96-Well Plate-Based Method for the Evaluation of P2X7 Receptor Activation Using YO-PRO-1 Fluorescent Dye. J. Biol. Methods.

[64] Lee D.Y., Lee S.Y., Yun S.H., Jeong J.W., Kim J.H., Kim H.W., Choi J.S., Kim G.-D., Joo S.T., Choi I. (2022). Review of the Current Research on Fetal Bovine Serum and the Development of Cultured Meat. Food Sci. Anim. Resour..

[65] Fadel L., Dacic M., Fonda V., Sokolsky B.A., Quagliarini F., Rogatsky I., Uhlenhaut N.H. (2023). Modulating Glucocorticoid Receptor Actions in Physiology and Pathology: Insights from Coregulators. Pharmacol. Ther..

[66] Jimeno B., Rubalcaba J.G. (2024). Modelling the Role of Glucocorticoid Receptor as Mediator of Endocrine Responses to Environmental Challenge. Philos. Trans. R. Soc. B.

[67] Pfaller A.M., Kaplan L., Carido M., Grassmann F., Díaz-Lezama N., Ghaseminejad F., Wunderlich K.A., Glänzer S., Bludau O., Pannicke T. (2024). The Glucocorticoid Receptor as a Master Regulator of the Müller Cell Response to Diabetic Conditions in Mice. J. Neuroinflammation.

[68] OCDE (2022). INERIS Les Informations de La Coordination Nationale Pour Les Lignes Directrices de l’OCDE [in French] 2022.

[69] Grimaldi M., Boulahtouf A., Toporova L., Balaguer P. (2019). Functional Profiling of Bisphenols for Nuclear Receptors. Toxicology.

[70] Chevolleau S., Debrauwer L., Stroheker T., Viglino L., Mourahib I., Meireles M.-H., Grimaldi M., Balaguer P., di Gioia L. (2016). A Consolidated Method for Screening the Endocrine Activity of Drinking Water. Food Chem..

[71] Mentor A., Wänn M., Brunström B., Jönsson M., Mattsson A. (2020). Bisphenol AF and Bisphenol F Induce Similar Feminizing Effects in Chicken Embryo Testis as Bisphenol A. Toxicol. Sci..

[72] Guo X., Wang H., Xu J., Hua H. (2022). Impacts of Vitamin A Deficiency on Biological Rhythms: Insights from the Literature. Front. Nutr..

[73] O’Connor C., Varshosaz P., Moise A.R. (2022). Mechanisms of Feedback Regulation of Vitamin A Metabolism. Nutrients.

[74] Koshy A.M., Mendoza-Parra M.A. (2023). Retinoids: Mechanisms of Action in Neuronal Cell Fate Acquisition. Life.

[75] Stunnenberg H.G. (1993). Mechanisms of Transactivation by Retinoic Acid Receptors. Bioessays.

[76] OECD (2021). Detailed Review Paper on the Retinoid System.

[77] Grignard E., Håkansson H., Munn S. (2020). Regulatory Needs and Activities to Address the Retinoid System in the Context of Endocrine Disruption: The European Viewpoint. Reprod. Toxicol..

[78] Balaguer P., Boussioux A.-M., Demirpence E., Nicolas J.-C. (2001). Reporter Cell Lines Are Useful Tools for Monitoring Biological Activity of Nuclear Receptor Ligands. Luminescence.

[79] Delfosse V., Huet T., Harrus D., Granell M., Bourguet M., Gardia-Parège C., Chiavarina B., Grimaldi M., Le Mével S., Blanc P. (2021). Mechanistic Insights into the Synergistic Activation of the RXR–PXR Heterodimer by Endocrine Disruptor Mixtures. Proc. Natl. Acad. Sci. USA.

[80] Engel S.M., Miodovnik A., Canfield R.L., Zhu C., Silva M.J., Calafat A.M., Wolff M.S. (2010). Prenatal Phthalate Exposure Is Associated with Childhood Behavior and Executive Functioning. Environ. Health Perspect..

[81] Mallozzi M., Bordi G., Garo C., Caserta D. (2016). The Effect of Maternal Exposure to Endocrine Disrupting Chemicals on Fetal and Neonatal Development: A Review on the Major Concerns. Birth Defects Res. Part C Embryo Today Rev..

[82] Paşca A.M., Sloan S.A., Clarke L.E., Tian Y., Makinson C.D., Huber N., Kim C.H., Park J.-Y., O’Rourke N.A., Nguyen K.D. (2015). Functional Cortical Neurons and Astrocytes from Human Pluripotent Stem Cells in 3D Culture. Nat. Methods.

[83] Acharya P., Choi N.Y., Shrestha S., Jeong S., Lee M. (2024). Brain Organoids: A Revolutionary Tool for Modeling Neurological Disorders and Development of Therapeutics. Biotechnol. Bioeng..

[84] Kiso-Farnè K., Yaoi T., Fujimoto T., Itoh K. (2022). Low Doses of Bisphenol A Disrupt Neuronal Differentiation of Human Neuronal Stem/Progenitor Cells. Acta Histochem. Cytochem..

[85] Yang L., Zou J., Zang Z., Wang L., Du Z., Zhang D., Cai Y., Li M., Li Q., Gao J. (2023). Di-(2-Ethylhexyl) Phthalate Exposure Impairs Cortical Development in hESC-Derived Cerebral Organoids. Sci. Total Environ..

[86] Koch K., Bartmann K., Hartmann J., Kapr J., Klose J., Kuchovská E., Pahl M., Schlüppmann K., Zühr E., Fritsche E. (2022). Scientific Validation of Human Neurosphere Assays for Developmental Neurotoxicity Evaluation. Front. Toxicol..

[87] Blum J., Masjosthusmann S., Bartmann K., Bendt F., Dolde X., Dönmez A., Förster N., Holzer A.-K., Hübenthal U., Keßel H.E. (2023). Establishment of a Human Cell-Based in Vitro Battery to Assess Developmental Neurotoxicity Hazard of Chemicals. Chemosphere.

[88] Hartmann J., Henschel N., Bartmann K., Dönmez A., Brockerhoff G., Koch K., Fritsche E. (2023). Molecular and Functional Characterization of Different BrainSphere Models for Use in Neurotoxicity Testing on Microelectrode Arrays. Cells.

[89] Neri C.R., Scapaticci S., Chiarelli F., Giannini C. (2022). Liver Steatosis: A Marker of Metabolic Risk in Children. Int. J. Mol. Sci..

[90] Castellana M., Donghia R., Guerra V., Procino F., Lampignano L., Castellana F., Zupo R., Sardone R., De Pergola G., Romanelli F. (2021). Performance of Fatty Liver Index in Identifying Non-Alcoholic Fatty Liver Disease in Population Studies. A Meta-Analysis. J. Clin. Med..

[91] American Liver Foundation (2024). American Liver Foundation Nonalcoholic Fatty Liver Disease (NAFLD) 2024.

[92] Teng M.L., Ng C.H., Huang D.Q., Chan K.E., Tan D.J., Lim W.H., Yang J.D., Tan E., Muthiah M.D. (2023). Global Incidence and Prevalence of Nonalcoholic Fatty Liver Disease. Clin. Mol. Hepatol..

[93] Cano R., Pérez J., Dávila L., Ortega Á., Gómez Y., Valero-Cedeño N., Parra H., Manzano A., Véliz Castro T., Albornoz M. (2021). Role of Endocrine-Disrupting Chemicals in the Pathogenesis of Non-Alcoholic Fatty Liver Disease: A Comprehensive Review. Int. J. Mol. Sci..

[94] Chen Y., Wang Y., Cui Z., Liu W., Liu B., Zeng Q., Zhao X., Dou J., Cao J. (2023). Endocrine Disrupting Chemicals: A Promoter of Non-Alcoholic Fatty Liver Disease. Front. Public Health.

[95] Lasch A., Marx-Stoelting P., Braeuning A., Lichtenstein D. (2021). More than Additive Effects on Liver Triglyceride Accumulation by Combinations of Steatotic and Non-Steatotic Pesticides in HepaRG Cells. Arch. Toxicol..

[96] Van Heemst D. (2024). The Ageing Thyroid: Implications for Longevity and Patient Care. Nat. Rev. Endocrinol..

[97] Mégier C., Dumery G., Luton D. (2023). Iodine and Thyroid Maternal and Fetal Metabolism during Pregnancy. Metabolites.

[98] Han Z., Chen L., Peng H., Zheng H., Lin Y., Peng F., Fan Y., Xie X., Yang S., Wang Z. (2023). The Role of Thyroid Hormone in the Renal Immune Microenvironment. Int. Immunopharmacol..

[99] Corsello S.M. (2020). Iodothyronine Deiodinases and Reduced Sensitivity to Thyroid Hormones. Front. Biosci..

[100] Köhrle J., Frädrich C. (2022). Deiodinases Control Local Cellular and Systemic Thyroid Hormone Availability. Free Radic. Biol. Med..

[101] Yuan S., Du X., Liu H., Guo X., Zhang B., Wang Y., Wang B., Zhang H., Guo H. (2023). Association between Bisphenol A Exposure and Thyroid Dysfunction in Adults: A Systematic Review and Meta-Analysis. Toxicol. Ind. Health.

[102] Rosen Vollmar A.K., Lin E.Z., Nason S.L., Santiago K., Johnson C.H., Ma X., Godri Pollitt K.J., Deziel N.C. (2023). Per- and Polyfluoroalkyl Substances (PFAS) and Thyroid Hormone Measurements in Dried Blood Spots and Neonatal Characteristics: A Pilot Study. J. Expo. Sci. Environ. Epidemiol..

[103] Coiffier O., Nakiwala D., Rolland M., Malatesta A., Lyon-Caen S., Chovelon B., Faure P., Sophie Gauchez A., Guergour D., Sakhi A.K. (2023). Exposure to a Mixture of Non-Persistent Environmental Chemicals and Neonatal Thyroid Function in a Cohort with Improved Exposure Assessment. Environ. Int..

[104] Schmutzler C., Gotthardt I., Hofmann P.J., Radovic B., Kovacs G., Stemmler L., Nobis I., Bacinski A., Mentrup B., Ambrugger P. (2007). Endocrine Disruptors and the Thyroid Gland—A Combined in Vitro and in Vivo Analysis of Potential New Biomarkers. Environ. Health Perspect..

[105] Joint Research Centre (2023). Validation of a Battery of Mechanistic Methods Relevant for the Detection of Chemicals That Can Disrupt the Thyroid Hormone System.

[106] Zhang J., Yang Y., Liu W., Schlenk D., Liu J. (2019). Glucocorticoid and Mineralocorticoid Receptors and Corticosteroid Homeostasis Are Potential Targets for Endocrine-Disrupting Chemicals. Environ. Int..

[107] Crouzet T., Grignard E., Brion F., Blanc E.B., Podechard N., Langouet S., Alonso-Magdalena P., Hubert P., Kim M.J., Audouze K. (2023). ReadEDTest: A Tool to Assess the Readiness of in Vitro Test Methods under Development for Identifying Endocrine Disruptors. Environ. Int..

[108] Scientific Committee on Consumer Safety (SCCS) (2021). SCCS Notes of Guidance for the Testing of Cosmetic Ingredients and Their Safety Evaluation—11th Revision.

[109] Kirkland D., Aardema M., Müller L., Makoto H. (2006). Evaluation of the Ability of a Battery of Three in Vitro Genotoxicity Tests to Discriminate Rodent Carcinogens and Non-Carcinogens II. Further Analysis of Mammalian Cell Results, Relative Predictivity and Tumour Profiles. Mutat. Res..

[110] Kirkland D., Reeve L., Gatehouse D., Vanparys P. (2011). A Core in Vitro Genotoxicity Battery Comprising the Ames Test plus the in Vitro Micronucleus Test Is Sufficient to Detect Rodent Carcinogens and in Vivo Genotoxins. Mutat. Res..

[111] (2014). Biological Evaluation of Medical Devices—Part 3: Tests for Genotoxicity, Carcinogenicity and Reproductive Toxicity 2014.

